# The Question of the Slaughter-Houses

**Published:** 1897-07-24

**Authors:** 


### The Question of the Slaughter-houses.

For some time past we have heard but little about slaughter-houses. The evils connected with these necessary but somewhat gruesome appendages of civilisation have long been well recognised. By almost general consent they used to be looked upon as nuisances. But in recent years their character has been much improved, so much so in fact that the slaughter-house question has in many places ceased to agitate the public mind.

There are signs, however, that the question of providing public abattoirs, and suppressing the private slaughter-houses which are now scattered in such numbers throughout London, will before long come to the front again, and that it will this time be discussed from a new point of view. It is not now as a means of mitigating a nuisance that a change will be advocated, but because under present conditions the proper inspection of the meat supply is an impossibility. The difficulties in the way of ensuring that none but healthy meat shall be sent to market are very great, while the importance of doing so is forcing itself upon us more and more every year.

Tuberculosis alone is a sufficiently serious matter. A very large proportion of our herds are admittedly infected, and if tuberculous meat is to be kept out of the market a vastly better system of inspection must be instituted than obtains at present. We cannot but feel, however, that the attention which has been directed to tuberculosis has rather tended to obscure the much larger question of the evils which accrue from the consumption of animals which, although not affected with such obvious disease, are still not in a condition of health. It is idle to expect a higher standard of morality from farmers than obtains in many other walks of life, and it has to be admitted that under the present system of inefficient inspection the slaughter-houses are sure to be used as the most handy means of turning doubtful cattle into cash.

The more strictly inspection is carried out at certain towns and in certain countries, the more do the feeble and the ailing beasts gravitate to those districts where the arrangements for detecting them are weak, especially to such places as from the multiplicity of their slaughter-houses offer the best chance of getting them through.

It is almost certainly an error to look upon the chance of catching the specific ailment from which an animal may have suffered as the only, or, indeed, as the principal danger arising from the ingestion of the flesh of diseased animals, and in our present ignorance of the real cause of various morbid conditions which are common in our midst the only safe course is to reject all meat, unless the animal from which it comes is healthy. This means careful, continuous, and minute inspection of all animals at the time of their being slaughtered and during the process of dressing the meat.

But at the present time we have in the metropolis nearly 500 separate slaughter-houses, mostly owned by private individuals, and the shallow pretence at inspection which goes on is a mere farce.

What is wanted is so to concentrate the places at which it is legal for cattle to be killed as to render inspection a reality. We have to recognise that the condition of the dressed meat is but an uncertain guide to the condition of the beast from which it is derived. It is far more important to see the animal cut up, and to observe the state of its entrails, than merely to look at the butcher's meat as it stands ready for the stalls, and this is just where the present system fails. It is quite impossible for the inspectors to know what is going on in the innumerable slaughter-houses scattered over London. But in fact so perfunctorily is the whole affair carried out that even at the docks, where one might expect to find some organised effort made to exclude disease, the arrangements are such as would excite the scorn of any German sanitary administrator. No doubt the butchers who are engaged as inspectors are very skilful men in their own line of business, and have a general knowledge of the coarse characteristics of healthy and unhealthy meat. But it is not by coarse characteristics that these matters should be decided, and there can be no possible doubt that if meat inspection is to give to the public all the benefits which it is capable of giving, the inspectors ought to be highly-trained men provided with proper scientific appliances for deciding the abstruse problems which must constantly come before them. But even from the ordinary butcher-inspector's point of view the present system is rendered farcical by the smallness of the staff employed. London cannot be protected from the evils arising from diseased meat by an inspector, however clever he may be, walking past a thousand carcases a day, and turning over a heap of offal without any means of assuring himself from which animal it has come.

The slaughter-house question cannot be allowed to remain much longer in its present anomalous condition. From the modern standpoint a far larger principal is involved than the mere abolition of a series of local nuisances. It is the public health that is in question, and the demand for the erection of public abattoirs and the abolition of private slaughter-houses comes on the ground that so only is it possible to ensure proper and continuous inspection, and to protect the public from the danger of eating the meat of diseased animals.

